# GIFT4 fusokine converts leukemic B cells into immune helper cells

**DOI:** 10.1186/s12967-016-0865-1

**Published:** 2016-04-27

**Authors:** Jiusheng Deng, Andrea Pennati, Jonathon B. Cohen, Yuanqiang Wu, Spencer Ng, Jian Hui Wu, Christopher R. Flowers, Jacques Galipeau

**Affiliations:** Department of Hematology and Medical Oncology, Winship Cancer Institute, Emory University, 1365B Clifton Road, Atlanta, GA 30322 USA; Department of Oncology, Lady Davis Institute for Medical Research, McGill University, Montreal, Canada

**Keywords:** Chronic lymphocytic leukemia, GIFT4 fusokine, T cells, Immunotherapy

## Abstract

**Background:**

Chronic lymphocytic leukemia (CLL) remains incurable with standard therapy, and is characterized by excessive expansion of monoclonal abnormal mature B cells and more regulatory immune properties of T cell compartment. Thus, developing novel strategies to enhance immune function merits further investigation as a possible therapy for CLL.

**Methods:**

We generated a fusion cytokine (fusokine) arising from the combination of human GM-CSF and IL-4 (named GIFT4). Primary CLL cells were treated with GIFT4 or GM-CSG and IL-4 in vitro. GIFT4-triggered STAT5 signaling in CLL cells was examined by Western blot. The phenotype and secretome of GIFT4-treated CLL cells (GIFT4-CLL cells), and the immune stimulatory function of GIFT4-CLL cells on autologous T cells were analyzed by flow cytometry and luminex assay.

**Results:**

GIFT4-CLL up-regulated the expression of co-stimulatory molecules CD40, CD80 and CD86 and adhesion molecule CD54. GIFT4-CLL cells secreted IL-1β, IL-6, ICAM-1 and substantial IL-2 relative to unstimulated CLL cells. GIFT4 treatment led to JAK1, JAK2 and JAK3-mediated hyper-phosphorylation of STAT5 in primary CLL cells, which is essential for GIFT4-triggered conversion of CLL cells. GIFT4-CLL cells directly propelled the expansion of autologous IFN-γ-producing CD314^+^ cytotoxic T cells in vitro, and that these could lyse autologous CLL cells. Furthermore, administration of GIFT4 protein promoted the expansion of human T cells in NOD-scid IL2Rγ^null^ immune deficient mice adoptively pre-transferred with peripheral blood mononuclear cells from subjects with CLL.

**Conclusion:**

GIFT4 has potent capability to converts primary CLL cells into APC-like immune helper cells that initiate a T cell driven anti-CLL immune response.

## Background

Chronic lymphocytic leukemia (CLL) is the most common leukemia in adults, and it is characterized by excessive expansion of monoclonal abnormal mature B cells in peripheral blood, lymph node, spleen and bone marrow [1], and remains incurable with standard therapy [2, 3]. T cells in patients with CLL have more regulatory immune response and less immune synapse interaction with antigen-presenting cells (APC) [4]. Thus, developing novel strategies to enhance immune function merits further investigation as a possible therapy for CLL. Emerging immunotherapies such as chimeric antigen receptor-modified T cells (CART) [5] and anti-CD20 monoclonal antibodies have shown improved outcomes for CLL management through direct attack on CLL B cells or activating complement membrane attack complex to lyse CLL B cells. However, B cell aplasia, decreased number of plasma cells, hypogammaglobulinemia and life-threatening cytokine release syndrome remain a challenge for CART therapy [6, 7]. Augmented T cell activation and immunity by CD40 ligation [8] and immune checkpoint blockade [3], and developing CLL vaccines [9, 10] are under clinical investigation, which provide alternative options for CLL immunotherapy.

We have developed a novel fusion cytokine (fusokine) named GIFT4 that is derived from GM-CSF and IL-4 [11], built upon the successful development of the GM-CSF and interleukin transgene platforms [12, 13]. GIFT4 protein has potent immune-stimulatory activity on B cells, and induces B cell proliferation through co-clustering of GM-CSF receptor (GM-CSFR) and IL-4 receptor (IL-4R) on the cell surface. GIFT4 treatment further programs naïve B cells into helper cells, which further license anti-tumor T cell response. Considering that CLL B cells express both IL-4R [14] and GM-CSFR [15], we tested whether GIFT4 could also have stimulatory effect on CLL cells, and reprogram the leukemic B cells into immune helper cells. Here we show that human GIFT4 stimulation converts primary CLL B cells into APC-like cells with up-regulated expression of co-stimulatory molecules CD40, CD80 and CD86 on their surface. GIFT4-converted CLL B cells (GIFT4-CLL cells) secreted IL-1β, IL-6, ICAM-1 and substantial IL-2, and primed autologous T cells from patients into IFN-γ-producing CD314^+^ CLL-killer cells.

## Methods

### Human subjects

Peripheral blood was collected from 12 subjects with CLL (seven males and five females) listed in Table 1 following informed consent, as per a protocol approved by the Emory University Institutional Review Board.

**Table 1:** Characteristics of CLL subjects

CLL subject number	Gender	Age	CD5^+^CD19^+^ CLL cells in PBMC (%)
1	M	78	93.2
2	F	74	87.6
3	M	64	93.8
4	M	57	81.6
5	M	72	86.1
6	M	38	84.2
7	F	56	95.6
8	F	75	82.6
9	F	52	81.0
10	F	76	87.4
11	M	54	94.7
12	M	62	86.0

### GIFT4 protein

The GIFT4 chimeric transgene was cloned from human GM-CSF and IL-4 cDNA (Invivogen), and the fusion protein was produced in bio-engineered 293T cells as previously described [11]. GIFT4 protein was concentrated and quantified by ELISA kit with anti-human GM-CSF antibodies (eBiosciences, San Diego, CA, USA).

### Cell culture

PBMC were isolated from peripheral blood of subjects with CLL using lymphocyte isolation solution (Mediatech). Primary CD5^+^CD19^+^ CLL cells were sorted from PBMC on a Becton–Dickinson FACS Aria Cell Sorter. T cells in PBMC were purified with T cell enrichment kits (StemCell). PBMC, primary CLL cells; T cells were cultured in complete RPMI-1640 medium (Corning, Manassas, VA, USA) for 5 days in presence of GIFT4 or recombinant GM-CSF and IL-4 control cytokines (2 ng/ml) (PeproTech). Alternatively, the treated CLL cells were washed with fresh RPMI-1640 medium, and cultured for additional 48 h. Cell culture supernatants were then collected and subjected to luminex assay with human 51plex cytokine polystyrene bead kit (Affymetrix, Santa Clara, CA, USA) in the Human Immunology Monitoring Center at Stanford University. For proliferation assay, PBMC or purified T cells from subjects with CLL were labeled with CFSE dye (Invitrogen, Eugene, OR, USA) following the manufacturer’s instruction, and cultured in complete RPMI 1640 medium for 5 days in presence of GIFT4 or GM-CSF and IL-4 (2 ng/ml). Autologous T cells were co-cultured with GIFT4-CLL cells or control cytokine-stimulated CLL cells (1:1 ratio) for 4 days. T cells were then purified with human T cell positive selection kit (StemCell) and re-cultured (10^5^/ml) in fresh RPMI-1640 medium for additional 48 h. The T cell culture supernatants were subjected to luminex assay.

### Flow cytometry

PBMC from subjects with CLL were stimulated with GIFT4 protein or GM-CSF and IL-4 (2 ng/ml) for 5 days. The cells were harvested and stained with APC-conjugated anti-human CD19 and PE-conjugated anti-human CD5 antibodies; or APC-conjugated anti-human CD3, then subjected to flow cytometry (FACS) on a BD FACSCanto II flow cytometer. Phenotype of GIFT4-CLL cells was profiled by FACS with a panel of B cell antibodies including anti-CD80 and CD86 (BD, San Diego, CA, USA). For analysis of cell–cell interaction between GIFT4-CLL cells and T cells, CFSE-labeled autologous T cells were co-cultured with GIFT4-CLL cells pre-treated with anti-human CD80 or CD86 neutralizing antibodies or isotype control (1 μg/ml) (BioLegend) for 5 days. T cell division was determined by FACS. For intracellular staining of IFN-γ, Granzyme B, and perforin, T cells were fixed and permeabilized with BD Cytofix/Cytoperm™ solution, followed by staining with PE-conjugated anti-human IFN-γ, granzyme B, perforin antibodies (BD). Alternatively, circulating human T cells in the peripheral blood of NSG immune deficient mice adoptively transferred with PBMC from CLL patients were profiled and counted by FACS, and analyzed with FlowJo 9.1 software.

### Luminex assay

The culture supernatants of GIFT4-treated primary CLL cells or control CLL cells were harvested, and the secretome of GIFT4-CLL cells was analyzed by luminex assay with human 51plex cytokine polystyrene bead kit as described [11].

### ELISA and western blot

IL-2 and IL-6 production by CLL cells was quantified with human IL-2 and IL-6 ELISA kit (eBiosciences, San Diego, CA, USA). CLL cells stimulated with human GIFT4 protein, GM-CSF and/or IL-4 (2 ng/ml) cytokines for 20 min in presence or absence of JAK inhibitors [11] were harvested, and lysed with protein lysate buffer supplemented with protease and phosphatase inhibitors as described [11]. STAT5 phosphorylation in the treated CLL cells was examined by Western blot with anti-pSTAT5 (Tyr694, D47E7) and anti-STAT5 antibodies (Cell Signaling, Boston, MA, USA).

### Cell apoptosis assay

GIFT4-CLL primed cytotoxic T cells (10^5^/ml) were co-cultured with primary CLL cells (10^5^/ml) in presence or absence of concanamycin (100 nM) (Sigma) for 24 h in a 96-well plate. The cells were collected and stained with APC-conjugated anti-human CD19 antibodies and Annexin V, then subjected to FACS analysis. Apoptotic cells were gated on Annexin V as previously described [16].

### CLL xenograft in NSG mice

PBMC from subjects with CLL (10^8^ cells/mouse) were adoptively transferred into NOD-scid IL2Rγ^null^ NSG mice [17] by intravenous injection. The mice were then treated with human GIFT4 protein (20 ng/mouse/day) or control cytokines for 6 days. On day 7, 100 μl of peripheral blood collected from each mouse were stained with anti-human CD3 antibody for 30 min, followed by addition of red blood cell lysis buffer (for 10 min) and AccuCheck Counting Beads (Invitrogen) to quantify circulating human T cell number as described [18, 19]. On day 30, human T cells in the peripheral blood of NSG mice were also analyzed by FACS with anti-human CD3 antibody (BD) before the mice were sacrified. All mice used were female (6–8 weeks old) purchased from Jackson Laboratory (Bar Harbor, ME, USA). The mice were maintained in compliance with an IACUC protocol approved by Emory University.

### Statistical analysis

Data were shown as mean ± SEM. *P* values were calculated using the one-way analysis of variance test. *P* value of less than 0.05 was considered significant (* *P* < 0.05; ** *P* < 0.01; *** *P* < 0.001).

## Results

### Human GIFT4 converts primary CLL B cells into antigen-presenting cell phenotype

**Fig. 1:**
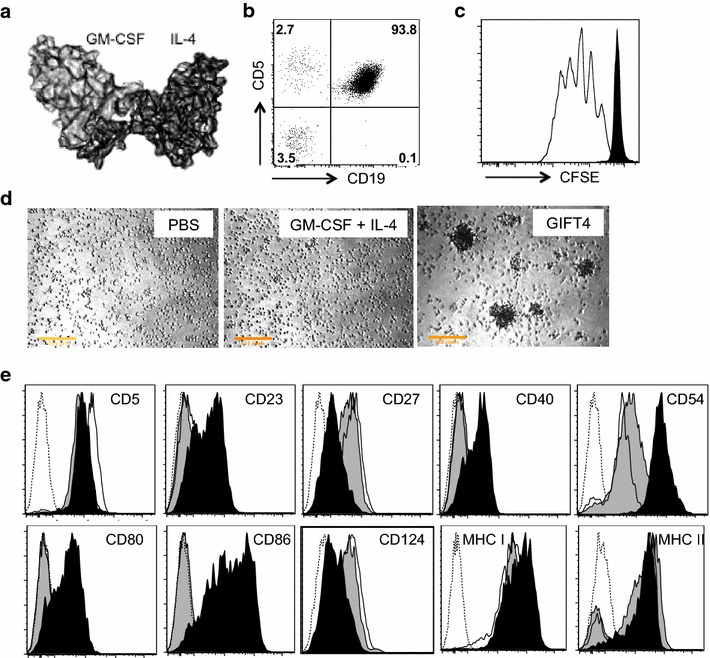
Phenotype of GIFT4-CLL cells. **a** Predicted 3D structure of GIFT4 protein. **b** A representative of CD19^+^CD5^+^ primary CLL cells in PBMC of subjects. **c** Purified normal human B cells (*White*) or CLL cells were labeled with CFSE dye and treated with GIFT4 protein for 5 days; cell proliferation was analyzed by FACS. **d** CLL cells treated with GIFT4, GM-CSF and IL-4 or PBS were photographed under a microscope (×10). **e** CLL cells treated with GIFT4 (*dark*), GM-CSF and IL4 (*gray*), or PBS (*white*) were subjected to FACS analysis with a *panel* of antibodies against B cell markers or with antibody isotype control (*Dash*). Data are representative of one of four repeated experiments using samples from subjects No. 1, 2, 3 and 4

We previously demonstrated that human GIFT4 could expand and reprogram normal human B cells into anti-tumor helper cells [11]. Thus we hypothesize that human GIFT4 protein (Fig. 1a) could have the capability to reprogram leukemic B cells into immune helper cells. To test the hypothesis, we isolated peripheral blood PBMC from the peripheral blood of subjects with CLL; the majority of peripheral blood mononuclear cells (PBMC) from the subjects we recruited are CD5^+^CD19^+^ leukemic B cells (Fig. 1b), ranged between 82.6 and 95.6 % with an average of 87.8 %. To check whether GIFT4 could promote the proliferation of CLL cells, the primary CLL B cells then were labeled with CFSE dye, then treated with human GIFT4 protein at a concentration of 2 ng/ml as previously described [11]. In contrast to GIFT4-induced expansion of normal human B cells [11], GIFT4 treatment did not trigger the proliferation of CLL B cells (Fig. 1c) although CLL B cells aggregated after GIFT4 stimulation (Fig. 1d), and did not activate peripheral T cells, NK cells or monocytes from subjects with CLL (Data not show). Profiling the surface molecules by FACS showed that GIFT4-CLL cells are CD23^+^, CD40^+^, CD80^+^, CD86^+^, MHCI^+^, and MHCII^+^, with enhanced expression of CD54 and down-regulation of CD27 and IL-4 receptor CD124 in comparison with control cytokine treatment (Fig. 1e). CLL cells treated with control cytokines or without treatment were absent of CD23, CD40, CD80 and CD86 (Fig. 1e).

**Fig. 2:**
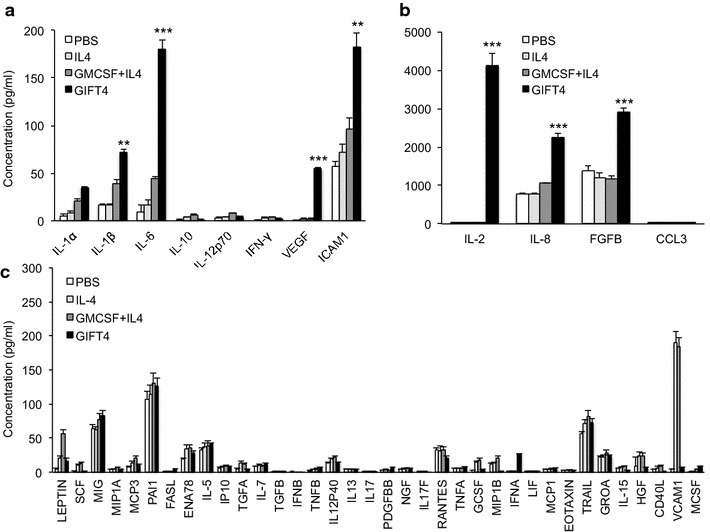
Secretome of GIFT4-CLL cells. Primary CLL cells were treated with GIFT4 (*Dark*), GM-CSF and IL-4 (*Dark gray*), IL-4 (*Light gray*) or PBS (*White*) for 5 days. The treated cells were harvested, washed, and re-culture for 24 h in fresh complete RPMI-1640 medium. The culture supernatants were collected and subjected to cytokine luminex assay with human 51plex cytokine polystyrene bead kit. **a**–**c** Cytokine and chemokine concentrations were calculated from three independent experiments using samples from subjects No. 2, 3 and 5

Primary human CLL cells have been shown to produce or express a similar level of 174 cytokines and cytokine receptors as normal B cells did, except low levels of IL-6 and eotaxin [20], and high levels of CXCR5 and CXCL13 [21]. We tested whether GIFT4 treatment of CLL cells would alter their secretome. Purified primary CLL cells were treated with GIFT4 protein or GM-CSF and IL-4 for 5 days. The cells were washed with fresh medium and cultured for additional 2 days. Luminex analyses on the culture supernatants showed that GIFT4-CLL cells produced significant amounts of immune-stimulatory cytokines and chemokines IL-6, IL-1β, VEGF, ICAM1 (Fig. 2a), and substantial amounts of IL-2, IL-8 and FGFB (Fig. 2b), in comparison with GM-CSF and IL-4 treated, or untreated CLL cells. Primary untreated CLL cells secrete low levels of cytokine such as TNF-α, IL-1β, IL-6 and IL-8 as previous described [22]. GIFT4-CLL cells secreted little of IL-10, GM-CSF, IFN-γ, and CCL3 (MIP1A) (Fig. 2a, b). There was no significant difference in the production of other cytokines and chemokines among GIFT4-CLL cells and CLL B cells treated with GM-CSF and IL-4 or PBS (Fig. 2c). However, there was a marked decrease of VCAM1 secretion by GIFT4-CLL cells compared with GM-CSF and IL-4 treated CLL cells (Fig. 2c).

### STAT5/JAK signaling is essential for the conversion of CLL cells by GIFT4 treatment

**Fig. 3:**
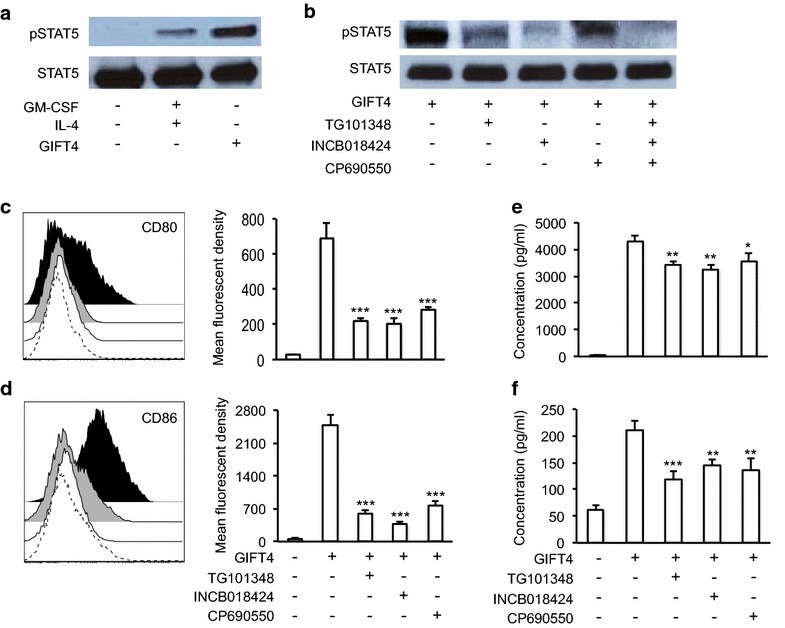
GIFT4-induced STAT5/JAK signaling in CLL cells. **a** Primary CLL cells were stimulated with GIFT4, GM-CSF and IL-4 or PBS for 20 min. The cells were harvested and lysed. Ten microgram of proteins in the cell lysate was subjected to Western blot analysis with anti-pSTAT5 or anti-STAT5 antibodies. **b** CLL cells were treated with GIFT4 protein in presence or absence of inhibitors for JAK2 (TG101348), JAK1/2 (INCB018424) or JAK3 (CP690550). The cell lysate was subject to Western blot analysis with anti-pSTAT5 and anti- STAT5 antibodies. **c**, **d** CLL cells were stimulated with GIFT4 protein for 5 days, supplemented with JAK inhibitors. CD80 (**c**) and CD86 (**d**) expression on CLL cells treated with GIFT4 protein (*Dark*), or GIFT4 with JAK2 inhibitor (*Dash*), JAK1/2 inhibitor (*White*), JAK3 inhibitor (*Gray*) was analyzed by FACS. Mean fluorescent density was also quantified. **e** and **f** Cytokine IL-2 and IL-6 secretion by the treated CLL cells was quantified by ELISA with anti-IL-2 and anti-IL-6 antibodies, and calculated from three repeated experiments using samples from subjects No. 6, 8 and 9

Cytokine-triggered early STAT signaling plays an important role in the regulation of gene expression and cell function [23]. In primary CLL cells, it has been reported that they deploy a constitutive increase of STAT1 and STAT3 phosphorylation [24, 25]. To explore the early STAT signaling events in CLL cells triggered by GIFT4 protein, we stimulated primary CLL cells with the fusokine or control cytokines. Western blot analysis showed that GIFT4 stimulation exclusively induced the hyper phosphorylation of STAT5 in comparison with GM-CSF and IL-4 treatment (Fig. 3a), but not of STAT1, STAT3 or STAT6 (data not shown). We further employed Janus protein tyrosine kinase 1 (JAK1), JAK2, and JAK3 specific inhibitors to examine whether JAK signaling is involved in GIFT4-triggered STAT5 hyper phosphorylation in CLL cells. Addition of JAK2, JAK1/2, or JAK3 specific inhibitors into the cell culture system significantly suppressed hyper phosphorylation of STAT5 in GIFT4-treated CLL cells (Fig. 3b). To determine the relevance of JAK signaling on the conversion of CLL cell phenotype by GIFT4 treatment, we utilized JAK inhibitors prior to GIFT4 stimulation. JAK1, JAK2 or JAK3 inhibitors robustly suppressed the GIFT4-induced expression of co-stimulatory molecules CD80 (Fig. 3c) and CD86 (Fig. 3d), but not CD40 (data not shown). To test whether GIFT4-induced STAT5/JAK signaling contributes to the cellular function of GIFT4-CLL cells on the production of immune stimulatory molecules, we used the same inhibitors to suppress JAK1, JAK2 and JAK3 signaling in the cell culture system respectively. We observed that inhibition of JAK signaling pathway significantly reduced the secretion of cytokine IL-2 (Fig. 3e) and IL-6 (Fig. 3f) by GIFT4-CLL cells.

### GIFT4-primed CLL cells expand autologous T cells in vitro and in vivo

**Fig. 4:**
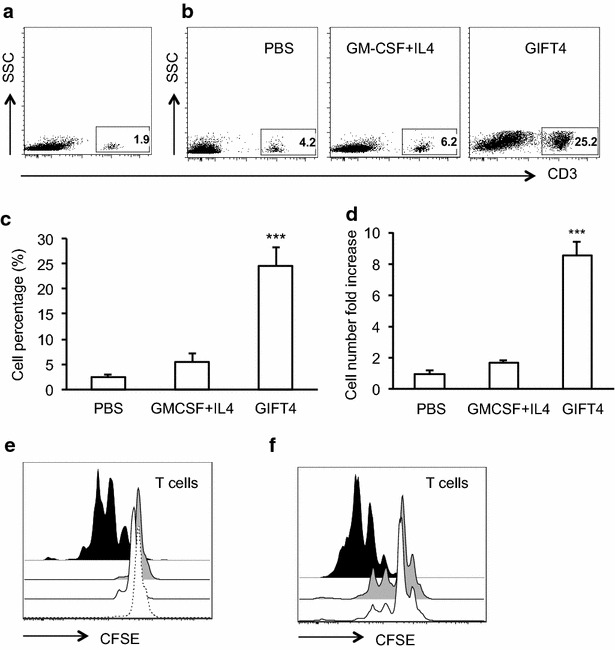
GIFT4 treatment induced the expansion of autologous T cells. PBMC were isolated from the peripheral blood of CLL patients, and stimulated with GIFT4, GM-CSF and IL-4 or PBS for 5 days. T cells in the PBMC before treatment (**a**), or after 5-day culture with GIFT4, GM-CSF and IL-4 or PBS (**b**) were profiled by FACS with anti-CD3 antibody; and T cell percentage and the absolute T cell number fold change were calculated from three independent experiments using samples from subjects No. 7, 10, and 11 (**c**, **d**). **e** CSFE-labeled autologous T cells were co-cultured with GIFT4-CLL cells (*Dark*), GM-CSF and IL-4 treated CLL cells (*Gray*), untreated CLL cells (*White*), or with GIFT4 protein (*Dash*) for 4 days. **f** Alternatively, anti-human CD80 (*Gray*), CD86 (*White*) neutralizing antibodies or isotype control (*Black*) were pre-incubated with GIFT4-CLL cells before co-cultured with CFSE-labeled autologous T cells. T cell division was analyzed by FACS (**e**, **f**)

**Fig. 5:**
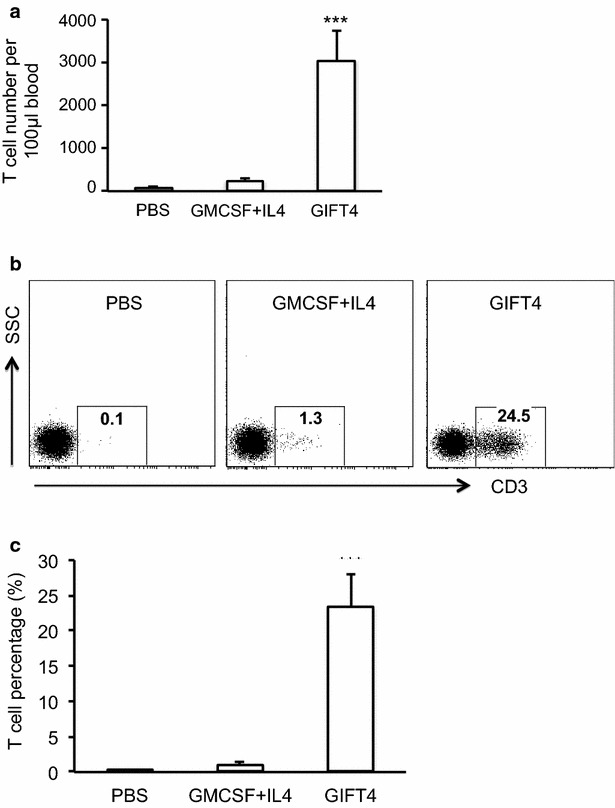
GIFT4 treatment expanded human T cells in NSG mice. PBMC from the CLL patients were adoptively transferred into NSG immune deficient mice, and treated with GIFT4, GM-CSF and IL-4 or PBS for 6 days. **a** On Day 7, peripheral blood were collected from the treated mice, and circulating human T cells in the blood were analyzed by FACS with anti-human CD3 antibody. T cell number per 100 μl blood was calculated based on bead counting. **b** On day 30, circulating human T cells in PBMC of treated mice were also profiled by FACS with anti-human CD3 antibody, and percentage of human T cells was presented (**c**). Data were from three independent experiments using samples from subjects No. 1, 3 and 7

To examine whether the conversion of primary CLL cells by GIFT4 treatment could lead to the gain-of-function immune-stimulatory activities of GIFT4-CLL cells on autologous T cells, we stimulated the PBMC from CLL patients with GIFT4, GM-CSF and IL-4, or PBS for 5 days. Profiling the cells by FACS showed that PBMC from subjects with CLL contained very low percentage of T cells (Fig. 4a). In comparison with CLL cells treated with control cytokine GM-CSF and IL-4 or without treatment, GIFT4-CLL cells induced robust proliferation of autologous T cells (Fig. 4b, c). The percentage of expanded autologous T cells in the co-cultured with CLL cells in presence of GIFT4 was 23.5 % on average, significantly higher than the original contents (Fig. 4a) or the ones in control treatments (Fig. 4c); with more than eightfold increase of absolute T cell number compared with the initial T cell number in the culture (Fig. 4d). To exclude the possibility that GIFT4 could have direct effect on T cells, we co-cultured CSFE-labeled autologous T cells with GIFT4-CLL cells or GM-CSF and IL-4 treated CLL cells, or with GIFT4 protein for 5 days. FACS analysis showed that GIFT4 stimulation alone without CLL cells could not induce T cell proliferation (Fig. 4e). To examine whether T cell expansion requires the cell–cell contact between GIFT4-CLL cells and autologous T cells, GIFT4-CLL cells were pre-incubated with anti-human CD80 or CD86 blocking antibodies. FACS analysis showed that blocking the co-stimulatory molecules inhibited T cell proliferation (Fig. 4f). To test whether human GIFT4-CLL cells could promote the expansion of T cells from subjects with CLL in vivo, we adoptively transferred CLL-containing PBMC into NOD-scid IL2Rγ^null^ (NSG) immune deficient mice, and treated the mice with GIFT4, GM-CSF and IL-4 or PBS. FACS analysis on the peripheral blood of the mice with anti-human CD3 antibody showed that GIFT4-treated CLL cells increased human T cell number in peripheral blood in comparison with GM-CSF and IL-4 or PBS control treatment (Fig. 5a). We also profiled human T cells in the treated NSG mice one month after treatment. In comparison with GM-CSF and IL-4 or PBS treatments, CLL cells treated with GIFT4 significantly enhanced the long-term survival and expansion of T cells from CLL patients in the NSG mice (Fig. 5b, c). We observed there was a graft versus host disease in the GIFT4-treated NSG mice but not in PBS- or GM-CSF and IL-4 treated groups, due to the cytotoxicity of GIFT4-CLL cell-primed human T cells.

### Human T cells primed by GIFT4-CLL cells are cytotoxic and kill primary CLL cells

**Fig. 6:**
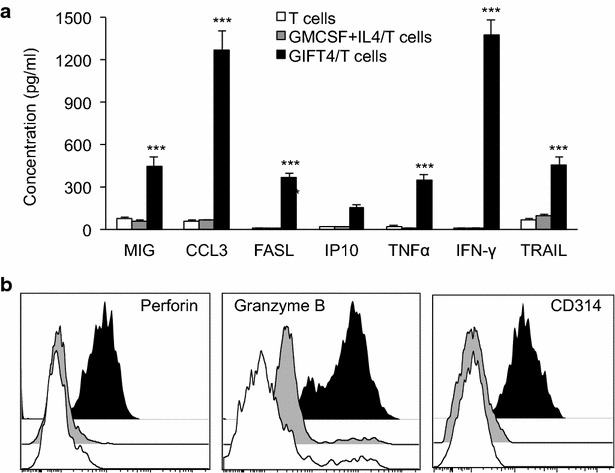
Secretome and cytotoxic factors of GIFT4-CLL cell primed T cells. **a** Autologous T cells primed by GIFT4-CLL cells (GIFT4/T cells) (*Dark*), GM-CSF and IL-4 treated CLL cells (GM-CSF and IL-4/T cells) (*Gray*) or PBS-treated CLL cells (T cells) (*White*) were purified with T cell isolation kits and re-cultured in fresh RPMI medium. The T cell supernatants were subjected to luminex assay, and cytokine/chemokine concentration was calculated from three independent experiments. **b** Cytotoxic factors perforin, granzyme B and CD314 produced or expressed by GIFT4-CLL cell primed T cells (GIFT4/T cells) (*Dark*), or by GM-CSF and IL-4/T cells (*Gray*) or control T cells (*White*) were analyzed by FACS with intracellular staining of perforin, granzyme B or surface staining of CD314. Data were represented from one of three repeated experiments using samples from subjects No. 9, 10 and 11

**Fig. 7:**
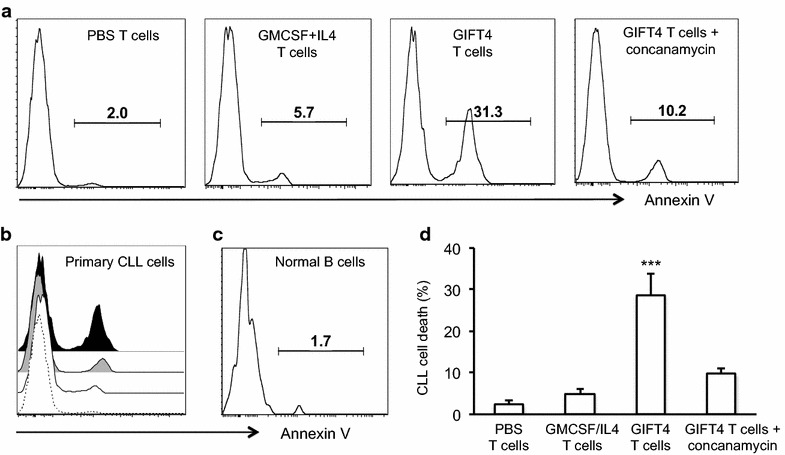
Killing of primary CLL cells by GIFT4-CLL cell primed T cells. **a**, **b** Primary CLL cells were co-cultured with normal T cells (PBS T cells), or with T cells primed by GM-CSF and IL-4 treated CLL cells (GMCS + IL4 T cells), or with GIFT4-CLL cell-primed autologous cytotoxic T cells (GIFT4 T cells) (1:1 ratio) in absence or presence of perforin inhibitor concanamycin for 24 h. The cells were then harvested and stained with anti-human CD19 antibody and Annexin V, and subjected to apoptotic analysis by FACS. **b** Combined histogram of Annexin V positive primary CLL cells. **c** Alternatively, normal B cells isolated from healthy subjects were co-cultured with GIFT4-CLL cell-primed T cells for 24 h before subjected to FACS analysis with Annexin V. **d** Percentage of apoptotic death of primary CLL cells in the treated groups was calculated from three independent experiments using samples from subjects No. 4, 8 and 12

To characterize GIFT4-CLL cell-primed T cells, the supernatant of the T cells were subjected to luminex assay. In comparison with T cells primed by GM-CSF and IL-4 or PBS treated CLL cells, GIFT4-CLL cell-primed T cells produced substantial amount of cytotoxic IFN-γ, MIP1A (CCL3), as well as FAS ligand, TRAIL, TNF-α, MIG (CXCL9), and IP-10 (Fig. 6a) relative to control. To further determine the cytotoxicity of GIFT4-CLL cell-primed T cells, we performed intracellular staining of cytotoxic factors perforin and granzyme B, and surface staining of tumor-killing molecule CD314 (NKG2D) on those T cells. FACS analysis showed that T cells primed by GIFT4-CLL cells are perforin^+^ and granzyme B^+^, and expressed CD314 (Fig. 6b). To test whether GIFT4-CLL cell-primed cytotoxic T cells could have the capability to directly kill primary CLL cells, we co-cultured primary CLL cells with autologous GIFT4-CLL cell-primed T cells. T cells primed by GM-CSF and IL-4 or PBS treated CLL cells served as control T cells. After 24-h culture, the primary CLL cells were profiled by FACS with anti-human CD19 antibody and Annexin V. Apoptotic assay showed that GIFT4-CLL cell-primed T cells significantly induced apoptosis of the primary CLL cells in comparison with control T cells (Fig. 7a, b), with 30.8 % of average apoptotic death induced by GIFT4-CLL cell-primed T cells (Fig. 7d). However, GIFT4-CLL cell-primed T cells did not induce apoptotic death of normal human B cells (Fig. 7c). Killing of primary CLL cells by cytotoxic T cells was found through perforin-mediated pathway [26]. Indeed, addition of concanamycin, a specific perforin blocker [26], significantly suppressed the killing of autologous primary CLL cells by GIFT4-CLL primed T cells (Fig. 7a, b, d).

## Discussion

In this study, we demonstrated that human GIFT4 can convert CLL B cells into immune-stimulatory helper cells, which function as APC and prime CLL-killing T cell response.

Primary CLL cells are monoclonal mature leukemic B cells that express CD5, CD19, CD20, IL-4 receptor (CD124), HLA-ABC and HLA-DR, but lack key T cell co-stimulatory molecules and adhesion molecules such as CD80, CD86, and CD54 [27]. It was reported that CD40 ligation of CLL B cells with CD40 ligand (CD154)-transduced human embryonic fibroblast cells up-regulated CD54, CD80 and CD86, but not CD40 [28]. In another study, transduction of adenovirus encoding chimeric CD154 augmented CLL cells to behave as APC [8]. Untreated CLL cells express similar high levels of TLR9 as normal B cells [29]. Activation of CLL cells with TLR9 ligand type B CpG oligodeoxynucleotides (CpG-ODN) resulted in significant increase of CD40, CD54, CD86, HLA-ABC and HLA-DR expression, but not CD80 [30]. Treatment of CLL cells with combined CpG-ODN and IL-21 also enhanced the expression of CD54 and CD80, with slight increase of CD40 and CD86 on the cell surface, enabling CLL B cells functioned as APC-like cells [31].

Unlike primary CLL cells, CD40- or TLR9-ligated CLL cells, or CpG/IL-21 treated CLL cells, GIFT4-CLL cells robustly up-regulate the expression of co-stimulatory molecules CD40, CD80 and CD86 and adhesion molecule CD54, which are likely essential surface factors for GIFT4-CLL cells functioning as APC to interact with T cells and prime T cell responses. Moreover GIFT4-CLL cells produce substantial amounts of IL-2, IL-8, FGFB, ICAM1, and IL-6, without significant production of GM-CSF, IFN-γ and CCL3. GIFT4-CLL cells are distinguished from our prior GIFT4-B cells that secrete GM-CSF and CCL3 [11] and different from CD40/OX40-ligated CLL cells that produce IFN-γ [28], or CpG/IL-21 treated CLL cells that do not produce IL-2, ICAM-1, IL-6 and FGFB but secrete granzyme B [31]. It has been reported that primary CLL cells produce CCL3 chemokine [20], however, we could not detect the chemokine in both untreated or GIFT4-treated CLL cells. Interesting, B cell receptor engagement with anti-IgM significantly enhanced chemokine CCL3 as well as CCL4 production by CLL cells [32]. Collectively, our data showed that GIFT4-converted CLL cells possess a unique phenotype and secretome, which facilitates GIFT4-CLL cells to function as potent APC.

JAK/STAT signaling plays an important role in the survival and surface molecule expression of CLL B cells [14, 15, 33, 34]. CLL cells express both IL-4R and GM-CSFR. The binding of IL-4R by IL-4 activates JAK signaling [34], and leads to the phosphorylation of STAT1, STAT5, and STAT6 that enhances the survival of CLL cells [14, 34]. Unlike normal human B cells, CLL cells only express the GM-CSFR α, but not β subunit [15, 34, 35]. GM-CSFR α was showed to link with the activation of STAT3 and to promote the survival of CLL cells [15]. GIFT4 has been previously shown to induce hyper phosphorylation of pan-STAT including STAT1, STAT3, STAT5 and STAT6 in normal B cells by clustering GM-CSFR and IL-4R on the cell surface and engagement of JAK1, JAK2 and JAK3 signaling [11]. Indeed, we observed that GIFT4 stimulation also induced hyper phosphorylation of STAT5 in CLL cells, which is involved in upstream collaborative signaling complex of JAK1, JAK2 and JAK3. GIFT4-triggered JAK/STAT5 signaling further contributes to both the expression of co-stimulatory molecules CD80 and CD86, and the production of T cell-promoting cytokines IL-2 and IL-6 by GIFT4-CLL cells. However, we did not detect hyper phosphorylation of STAT1, STAT3 and STAT6 in CLL cells by GIFT4 exposure. Whether the lack of GM-CSFR β is linked to the absence of hyper phosphorylation of STAT1, STAT3 and STAT6 in CLL cells by GIFT4 stimulation remains to be determined.

Modified B cells have been utilized for cancer immunotherapy [36, 37]. Activated B cells acting as APC can elicit anti-tumor T cell response [11, 38, 39] or possess tumor-killing ability independently or through anti-tumor antibody production [40, 41]. Previous studies have also shown that in vitro modified CLL cells hyper-expressing adhesion molecules B7-1, ICAM-1 and LFA-3 [42], CD40 ligand [43] or both CD40 ligand and IL-2 [44] may enhance effective T cell responses against CLL cells. Vaccination of whole CLL cells admixed with irradiated GM-CSF-secreting K562 bystander cells also promoted the expansion of IFN-γ^+^ leukemic-reactive T cells against CLL in patients after hematopoietic stem cell transplantation [9]. GIFT4-CLL cells appear to have profound antigen presentation properties that may improve upon these approaches. GIFT4-converted CLL cells not only express immune co-stimulatory molecules CD40, CD80, CD86 and adhesion molecule ICAM-1 but also secrete immune-stimulatory cytokines IL-2 and IL-6, and have the potent ability to promote the expansion of autologous T cells. Those T cells produce cytotoxic factors such as IFN-γ, perforin, granzyme B, TRAIL, FAS ligand, CD314, and can directly kill primary CLL cells through perforin-mediated pathway. To our knowledge, this is the first report that primary CLL cells from subjects with CLL can be reprogrammed to anti-CLL immune helper cells.

## Conclusions

GIFT4 fusokine has potent capability to reprogram CLL cells into APC-like effectors, expressing co-stimulatory molecules CD40, CD80, CD86, and adhesion molecules CD54. GIFT4-CLL cells secreted immune stimulatory cytokines IL-1β, IL-6, ICAM-1 and substantial IL-2, and prime autologous T cells into IFN-γ-producing CD314^+^ CLL-killer cells. The exclusive characteristics and the unique functions of GIFT4 and GIFT4-CLL cells support the notion that GIFT4 fusokine and GIFT4-CLL cells could be utilized as novel therapeutics for CLL immunotherapy.
